# Two-decade *in-situ* oceanographic and meteorological observations from Ieodo Ocean Research Station in the northern East China Sea

**DOI:** 10.1038/s41597-026-06769-4

**Published:** 2026-02-09

**Authors:** Go-Un Kim, Yongchim Min, Seung-Woo Lee, Hyoeun Oh, Jongmin Jeong, Juhee Ok, Jaeik Lee, Su-Chan Lee, In-Ki Min, Euiyoung Jeong, Kwang-Young Jeong, Hyunsik Ham, Jin-Yong Jeong

**Affiliations:** 1 Korea Institute of Ocean Science and Technology, Busan, South Korea; 2 Center for Climate Physics, Institute for Basic Science, Busan, South Korea; 3 Pusan National University, Busan, South Korea; 4 Ocean Research Division, Korea Hydrographic and Oceanographic Agency, Busan, South Korea

## Abstract

The East China Sea (ECS) is a climate-sensitive region experiencing rapid oceanic and ecological changes, with warming rates approximately twice those of the global average. Sustained long-term observations are essential to detect and understand these changes. The Ieodo Ocean Research Station (I-ORS), established in June 2003 on the northern ECS continental shelf, serves as the first continental shelf platform in the global ocean observation network OceanSITES. Over two decades (2004–2023), I-ORS has continuously monitored oceanographic and meteorological variables in real time. Here, we present quality-controlled hourly datasets, including water temperatures at 5, 21, and 38 m, air temperature and pressure, winds, relative humidity, and precipitation, derived through systematic processing. Comprehensive validation demonstrates the dataset’s quality, its capability to resolve variability from diurnal to decadal timescales, and its regional representativeness across the northern ECS. This openly available dataset supports studies of air-sea interactions and climate change impacts, with applications in forecasting, early warning systems, and disaster management for the region.

## Background & Summary

Global warming has led to marked changes in marine environments worldwide. The East China Sea (ECS) has emerged as a climate change hotspot region^1–3^, showing heightened vulnerability to these changes. In the early 21^st^ century, sea surface temperature (SST) in the ECS has increased by 0.83 °C per decade, approximately twice the rate in the Pacific Ocean (0.42 °C per decade)^4^. Accordingly, chlorophyll-a concentrations—a key indicator of phytoplankton biomass and primary production—have declined^1–3^, which could ultimately affect marine ecosystem stability in the ECS. Under continued climate change scenarios, these impacts are projected to intensify and become potentially irreversible^5–7^, posing serious threats to fisheries and food security. Despite the urgent need to understand and address climate change impacts on the ECS, sustained *in-situ* observations remain scarce in this region^8^. Detecting and attributing these changes requires long-term continuous observations. Such observations are uniquely valuable for climate research, as they capture both short-term variability and long-term trends that satellite data and numerical models alone cannot resolve. Therefore, sustained real-time monitoring platforms are essential to complement existing satellite and reanalysis products.

**Fig. 1:**
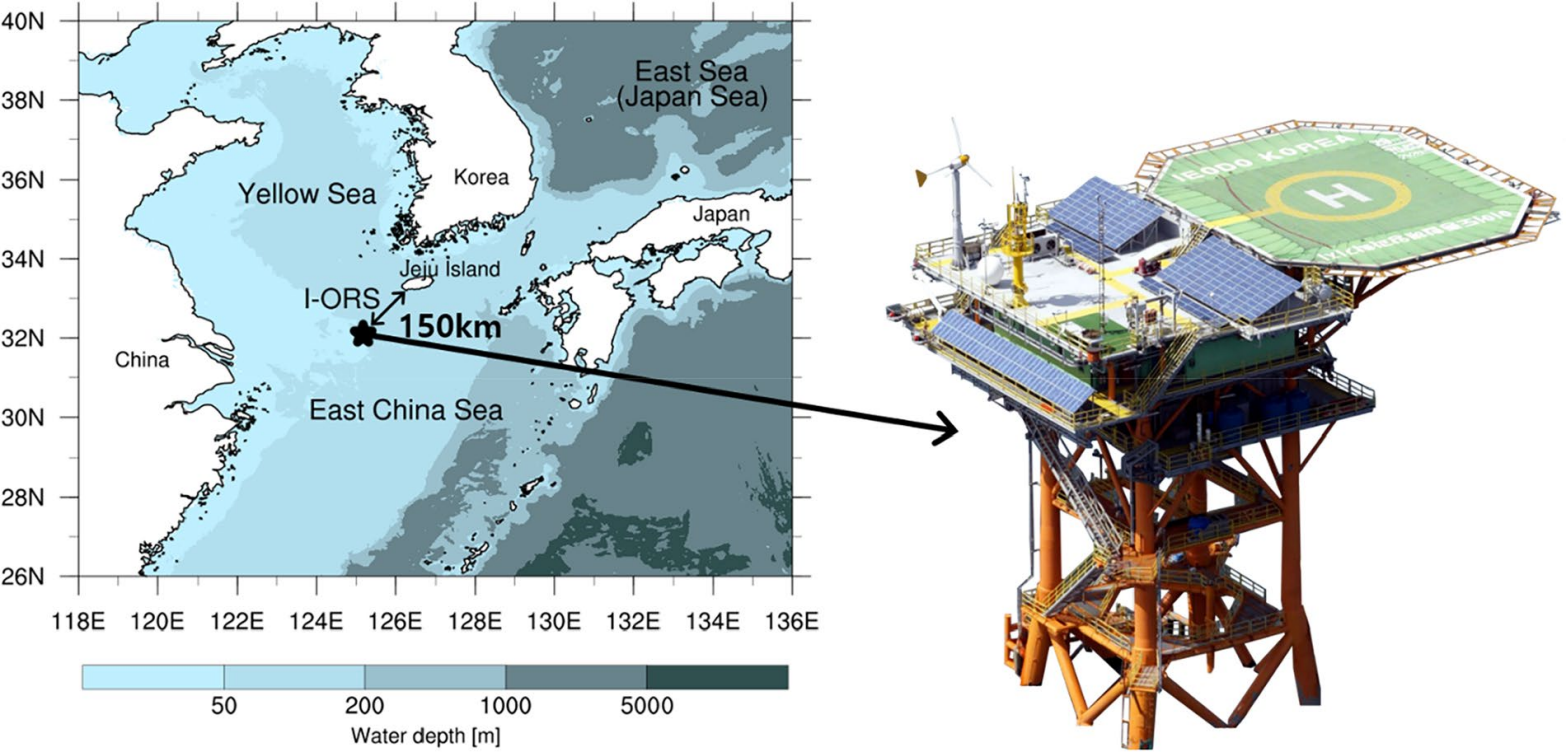
Location of the Ieodo Ocean Research Station (I-ORS; star marker) and photo of its structure.

This study introduces the Ieodo Ocean Research Station (I-ORS), an ocean observation platform providing long-term oceanographic and meteorological data for understanding climate change impacts and ocean-atmosphere phenomena in the northern ECS^9,10^ (Fig. 1). The I-ORS is strategically positioned at 32.12°N, 125.18°E, ~150 km southwest of Jeju Island, at the intersection of multiple water masses including the northward-flowing Kuroshio Current, the southward-extending Yellow Sea Cold Water Mass, and the seasonally varying Changjiang Diluted Water. This offshore location minimizes terrestrial influences while enabling comprehensive monitoring of ocean-atmosphere interactions. The station also lies along a major typhoon pathway from the western Pacific toward East Asia, providing an advantageous location for observing tropical cyclone characteristics.

**Fig. 2:**
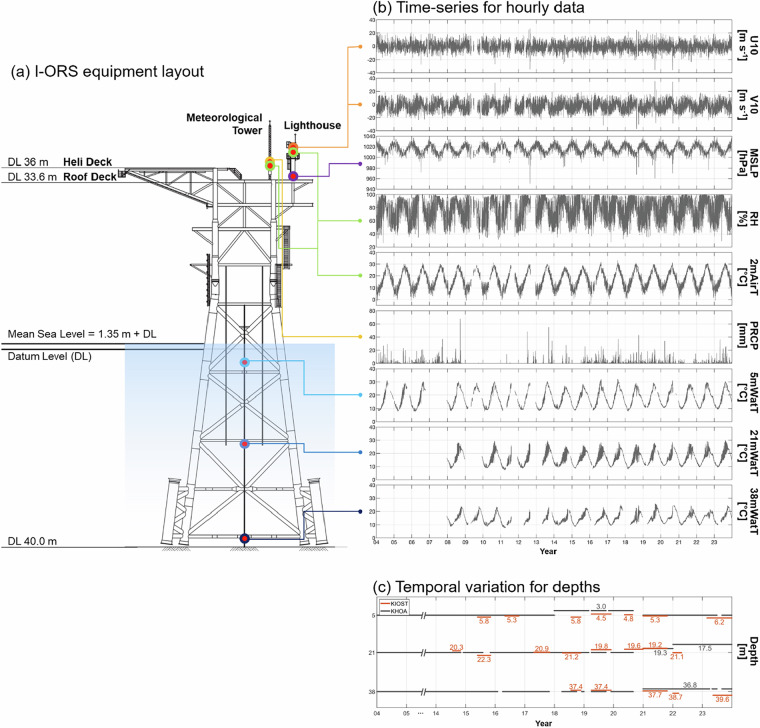
The I-ORS monitoring system. (**a**) equipment layout, (**b**) the corresponding hourly time-series data monitored by these instruments, and (**c**) temporal variations in water temperature depths during 2004–2023. The measurement depths at 5, 21, and 38 m were subject to slight variations due to instrument changes.

The I-ORS is constructed on Ieodo, a submerged reef with its summit at ~4.6 m below mean sea level, where the water depth is approximately 40 m. The platform is a jacket-type steel structure extending from 40 m below datum level (DL; approximate lowest low water) to 36 m above it, with a total structural height of 76 m (Fig. 2a). Since its completion in June 2003, I-ORS has continuously measured a wide range of oceanographic and meteorological parameters in real time for over 20 years, supported by a stable power supply from solar and diesel generators and robust communication systems via long-term evolution and satellite links. Unlike most platforms in the global ocean observation network OceanSITES that focus on deep-sea environments (>2,000 m in depth), I-ORS became the first continental shelf observation platform registered in the network in 2018. This unique position offers valuable observational capabilities in shallow-water environments sensitive to coastal processes and regional climate variability in the northern ECS.

These capabilities have resulted in numerous significant research contributions. I-ORS observations have enabled studies of long-term climate trends (temperature changes^11^ and sea level rise^12^), extreme events (typhoons^13–16^ and marine heatwaves^17–19^), and seasonal phenomena (Changjiang Diluted Water^10,18,20,21^, East Asian monsoons^22,23^, and sea fog^24^). Studies have also revealed insights into oceanic internal waves^25,26^ and fishing activity-related hydraulic characteristics in the northern ECS^27^. Designed for a lifespan exceeding 50 years, I-ORS will continue producing valuable observations for at least three more decades. These ongoing observations will contribute to improved forecasting, disaster response, and sustainable environmental management of the ECS region.

## Methods

### Data collection

The I-ORS observational data were collected over a 20-year period (2004–2023) from two sources: the Korea Hydrographic and Oceanographic Agency (KHOA; 10.17882/108509^28^; 10.17882/108530^29^; 10.17882/85130^30^; 10.17882/92401^31^; 10.17882/92402^32^; 10.17882/97666^33^; 10.17882/103952^34^) and the Korea Institute of Ocean Science and Technology (KIOST; 10.22808/DATA-2024-6^35^). Most variables were recorded at 10-min intervals in Coordinated Universal Time (UTC), with precipitation at hourly intervals. Oceanographic variables from KHOA and KIOST include water temperature at different depths. KHOA data were primarily used, complemented by KIOST data (2014–2023) during periods with data gaps or sensor errors. These data were obtained using three conductivity-temperature-depth (CTD) sensors—CT3919, SBE37, and RBR Concerto—at nominal depths of 5, 21, and 38 m below mean sea level from the moored CTD array (Fig. 2a and 2b). Measurement depths varied slightly during the observation period due to instrument replacement and maintenance (Fig. 2c). Meteorological variables from KHOA include air temperature and pressure, zonal and meridional winds, relative humidity, and precipitation. These were measured using instruments installed across the roof deck of the platform at 33.6 m above DL (Fig. 2a and 2b): two HMP155 thermo-hygrometer sensors at the lighthouse (42.1 m) and meteorological tower (36.6 m) for air temperature and relative humidity; two 05106 anemometer sensors at the lighthouse (42.9 m) for zonal and meridional winds; a PTB210B barometer sensor in a box on the roof deck (34.6 m) for air pressure; and a bucket type rain gauge at the meteorological tower (37 m) for precipitation. Table 1 and Fig. 2 present detailed sensor specifications, instrument locations, and temporal variations in measurement depths.

**Table 1:** Observation variable (unit), sensor, model, manufacturer, and accuracy at the I-ORS.

Variable (unit)	Sensor	Model	Manufacturer	Accuracy
Water temperatures at 5, 21, and 38 m (°C)	CTD	CT3919 SBE37 RBR Concerto	Aanderaa Seabird RBR	± 0.1 °C ± 0.002 °C ± 0.002 °C
Air temperature (°C) Relative humidity (%)	Thermo-hygrometer	HMP155	VAISALA	Temperature-dependent: ± (0.226−0.0028 × temperature) °C at −80 to +20 °C ± (0.055+0.0057 × temperature) °C at +20 to +60 °C Humidity-dependent: ± 1 %RH (0–90 %RH) at +15 to +25 °C ± 1.7 %RH (90–100 %RH) at +15 to +25 °C ± (1.2 + 0.012 × reading) %RH at +40 to +60 °C ± (1.0 + 0.008 × reading) %RH at −20 to +40 °C ± (1.2 + 0.012 × reading) %RH at −40 to −20 °C ± (1.4 + 0.032 × reading) %RH at −60 to −40 °C
Zonal wind (m s^−1^) Meridional wind (m s^−1^)	Anemometer	05106	RMYoung	± 0.3 m s^−1^ (0.6 mph) or 1% of reading
Air pressure (hPa)	Barometer	PTB210B	VAISALA	± 0.20 hPa
Precipitation (mm)	Rain gauge	Bucket type	ELP	±1% (50 mm h^−1^)

### Data processing

**Fig. 3:**
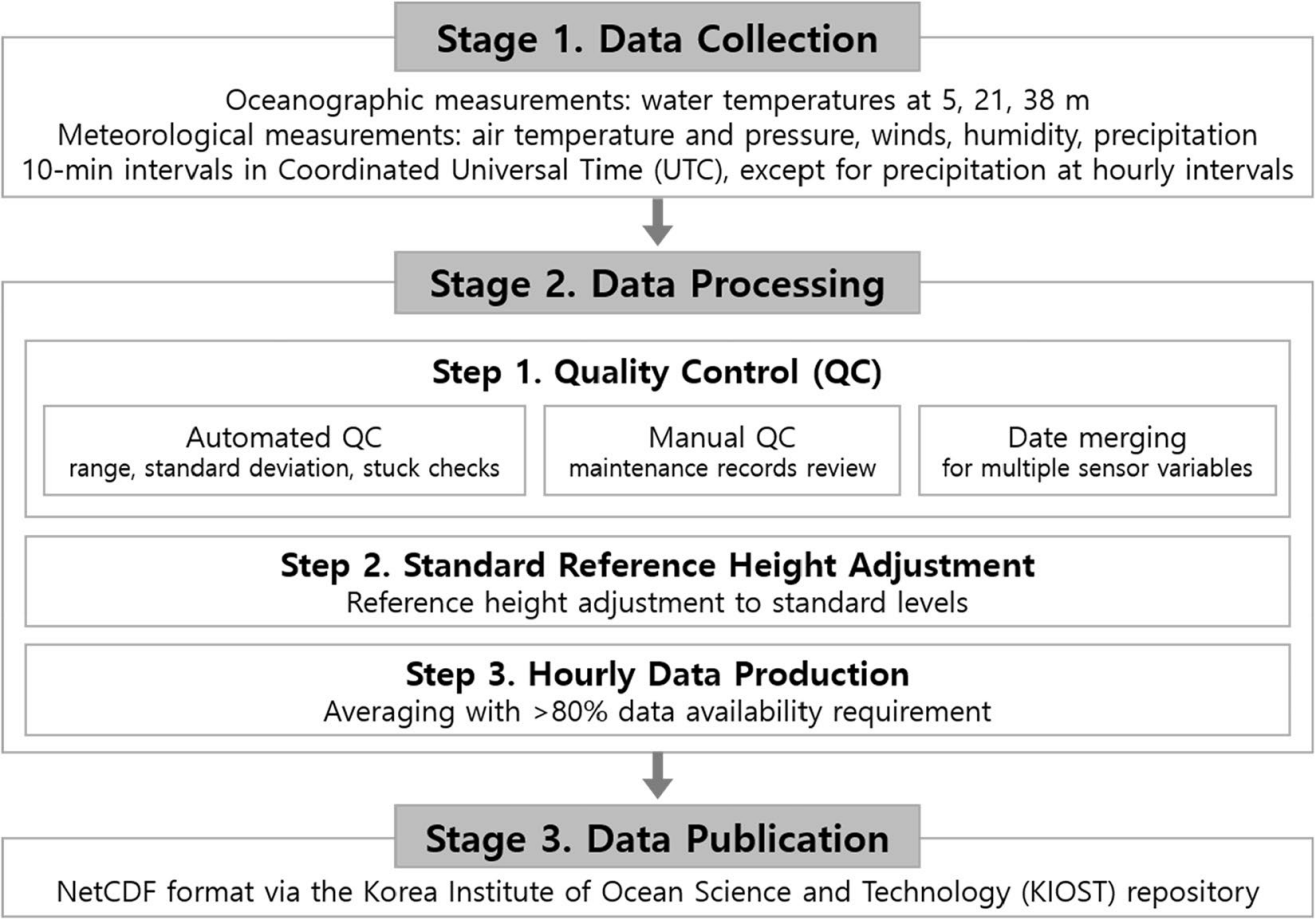
Three-stage workflow for the I-ORS data.

I-ORS observations undergo three major workflow stages (Fig. 3): Stage 1 (data collection), Stage 2 (data processing), and Stage 3 (data publication). Here we focus on Stage 2. Raw I-ORS data were subject to errors due to various environmental and technical constraints, including severe weather, biofouling-induced sensor degradation, power supply instability, sensor interference, and network communication issues. To ensure integrity and high-quality of observational data, we implemented a systematic three-step procedure: step (1) quality control (QC) and merging of multiple sensor records, step (2) conversion to standard heights, and step (3) aggregation of validated 10-min records into hourly values.

*QC*. This study employed a two-part QC system for the observational data^9,36^. Part 1: automated QC identified outliers using three methods: (1) range check, which examined whether values exceeded predefined thresholds for each variable; (2) standard deviation check, which detected values that exceeded statistical mean variability using a moving window approach; and (3) stuck check, which identified anomalous sequences of identical values inconsistent with natural variability. Part 2: manual QC involved reviewing field maintenance logs to identify events affecting data quality, such as sensor cleaning, inspection, and replacement. Cross-sensor consistency checks were also performed between multiple sensors with related oceanic-atmospheric variables. For variables measured by multiple sensors (zonal and meridional winds, air temperature, relative humidity, and water temperatures at different depths), validated records were merged into a single composite time series after QC.

#### Standard reference height adjustment

To facilitate interoperability, comparability, and homogeneity across datasets and research communities, all QC-processed data were adjusted to standard reference heights following the established guidelines^37,38^.

Wind components were converted from the measurement height of 42.9 m (${U}_{42.9m}$; m s^−1^) to the standard 10 m reference height (${U}_{10m}$; m s^−1^) using the logarithmic wind profile established through Monin-Obukhov similarity theory under neutral stability conditions^37,39^. Under this assumption, wind (${U}_{z}$; m s^−1^) at height ($Z$; m) is expressed as:

$${U}_{z}=\left({u}_{* }/\kappa \right)\mathrm{ln}(z/{z}_{0})$$

where ${u}_{* }$ is friction velocity (m s^−1^), ${z}_{0}$ is surface roughness (m), and the von Karman constant is $\kappa$ = 0.4. The 10 m wind was calculated as:

$${U}_{10m}={U}_{42.9m}\frac{\mathrm{ln}(10/{z}_{0})}{\mathrm{ln}(42.9/{z}_{0})}$$

where the friction velocity is ${u}_{* }=\,\kappa {U}_{42.9m}/\mathrm{ln}(42.9/{z}_{0})$ m s^−1^, and the surface roughness is ${z}_{0}=\,$5 × 10^−4 ^m, corresponding to an intermediate value between typical roughness lengths observed in open ocean and coastal regions^22,40–42^.

Air temperature was adjusted from the averaged measurement height of 39.4 m (${T}_{39.4m}$; °C) to the standard 2 m reference height (${T}_{2m}$; °C) using a linear lapse-rate relationship^37,38,43^. The molecular-scale temperature (${T}_{M}$; °C) as a function of geopotential height ($H$; m) is given by:

$${T}_{M}={T}_{M,b}+{L}_{M,b}(H-{H}_{b})$$

where ${T}_{M,b}$, ${L}_{M,b}$, and ${H}_{b}$ are the molecular-scale temperature (°C), molecular-scale lapse rate (°C km^−1^), and geopotential height (m) at the base of a specific atmospheric layer, respectively. Subscript *b* denotes the base of a specific atmospheric layer and subscript *M* denotes the molecular-scale value with height dependence. Applying the standard atmospheric lapse rate (${L}_{M,b}=$ −0.0065 °C m^−1^), air temperature at 2 m was estimated as:

$${T}_{2m}={T}_{39.4m}-0.0065(2-39.4)$$

where the measurement height is ${H}_{b}=$ 39.4 m and the standard reference height is $H=$ 2 m.

Air pressure was reduced from the measurement height of 34.6 m (${P}_{34.6m}$; hPa) to mean sea level (${P}_{meansealevel}$; hPa) using the integrated hydrostatic equation under the assumption of constant air density, expressed as:

$${P}_{2}-{P}_{1}=-\rho g({Z}_{2}-{Z}_{1})$$

where $P$ is pressure (Pa), $Z$ is height (m), the air density is $\rho =$ 1.225 kg m^−3^, and the standard gravity is $g=$ 9.8 m s^−2^. Subscript *1* and *2* denote two different vertical levels. The mean sea level pressure was derived as:

$${P}_{{mean}{sea}{level}}={P}_{34.6m}-0.12(0-34.6)$$

where the measurement height is ${Z}_{1}=$ 34.6 m and the reference height is ${Z}_{2}=$ 0 m.

Hereinafter, variable names are denoted as follows: water temperatures at 5, 21, and 38 m (5mWatT, 21mWatT, and 38mWatT); air temperature at 2 m (2mAirT); 10 m zonal and meridional winds (U10 and V10); mean sea level pressure (MSLP); relative humidity (RH); and precipitation (PRCP).

#### Hourly data production

Following World Meteorological Organization guidelines on the calculation of climate normals^44^, we calculated hourly data for oceanographic and most meteorological parameters by arithmetic averaging when at least 80% of the 10 min observations (i.e., five out of six observations) were available within each hour. Hours with data availability <80% were flagged as −99999 (missing values).

## Data Records

All I-ORS hourly datasets are openly available under CC BY 4.0 via the KIOST repository (10.22808/DATA-2025-2^45^; Stage 3 in Fig. 3). The data are provided in NetCDF (.nc) format, with filenames structured as follows: < site name > _ < start year > _ < end year > _ < variable group > _ < time resolution > .nc (Table 2). In this structure, the site name is I-ORS, start and end years represent the observation period (2004–2023), variable group is categorized as either ocean (OCN) or atmosphere (ATM), and time resolution is hourly. Specifically, oceanic variables (i.e., 5mWatT, 21mWatT, and 38mWatT) are stored in I-ORS_2004_2023_OCN_1hr.nc, while atmospheric variables (i.e., 2mAirT, U10, V10, MSLP, RH, and PRCP) are included in I-ORS_2004_2023_ATM_1hr.nc.

**Table 2:** File structure of I-ORS_2004_2023_OCN_1hr.nc and I-ORS_2004_2023_ATM_1hr.nc.

File name	Variable name	Description
I-ORS_2004_2023_OCN_1hr.nc	TIME	Time
	LATITUDE	Latitude
	LONGITUDE	Longitude
	5mWatT	5 m water temperature (°C)
	21mWatT	21 m water temperature (°C)
	38mWatT	38 m water temperature (°C)
I-ORS_2004_2023_ATM_1hr.nc	TIME	Time
	LATITUDE	Latitude
	LONGITUDE	Longitude
	2mAirT	2 m air temperature (°C)
	U10	10 m zonal wind (m s^−1^)
	V10	10 m meridional wind (m s^−1^)
	MSLP	Mean sea level pressure (hPa)
	RH	Relative humidity (%)
	PRCP	Precipitation (mm)

## Technical Validation

Although *in-situ* observations generally provide highly reliable measurements, systematic validation was necessary to quantitatively assess the quality of the processed I-ORS dataset. This study evaluated the I-ORS time series against multiple reanalysis datasets at hourly and daily timescales from 2004 to 2023, including daily water temperatures (0.5, 21, and 40 m) from the Copernicus Marine Environment Monitoring Service’s Global Ocean Reanalysis and Simulation version 12 (GLORYS12; 10.48670/moi-00021^46^) with a horizontal resolution of 1/12°, daily SST from the National Oceanic and Atmospheric Administration’s Optimal Interpolation Sea Surface Temperature version 2 (OISST V2; https://psl.noaa.gov/data/gridded/data.noaa.oisst.v2.highres.html^47^) with a spatial grid of 1/4°, daily precipitation from the Multi-Source Weighted-Ensemble Precipitation version 2 (MSWEP V2; https://www.gloh2o.org^48^) with spatial resolution of 0.1°, and other hourly atmospheric variables from the European Centre for Medium-Range Weather Forecasts Reanalysis version 5 (ERA5; 10.24381/cds.adbb2d47^49^, 10.24381/cds.bd0915c6^50^) with 0.25° × 0.25° latitude–longitude grid. Given the absence of other observational platforms in spatial proximity to the offshore I-ORS location, we extracted data from the nearest reanalysis grid points (ERA5: 32°N, 125.25°E; MSWEP V2: 32.15°N, 125.15°E; GLORYS12: 32.083°N, 125.167°E) to the I-ORS location (32.12°N, 125.18°E) for a point-to-point comparison. The validation encompasses four analyses: hourly diurnal cycle assessment, daily comparison with reanalysis products, spatial representativeness across the northern ECS, and long-term trend detection. These analyses confirm this dataset’s quality, its capability to resolve variability from diurnal to decadal timescales, and its regional representativeness across the northern ECS.

### Hourly-scale diurnal variability

**Fig. 4:**
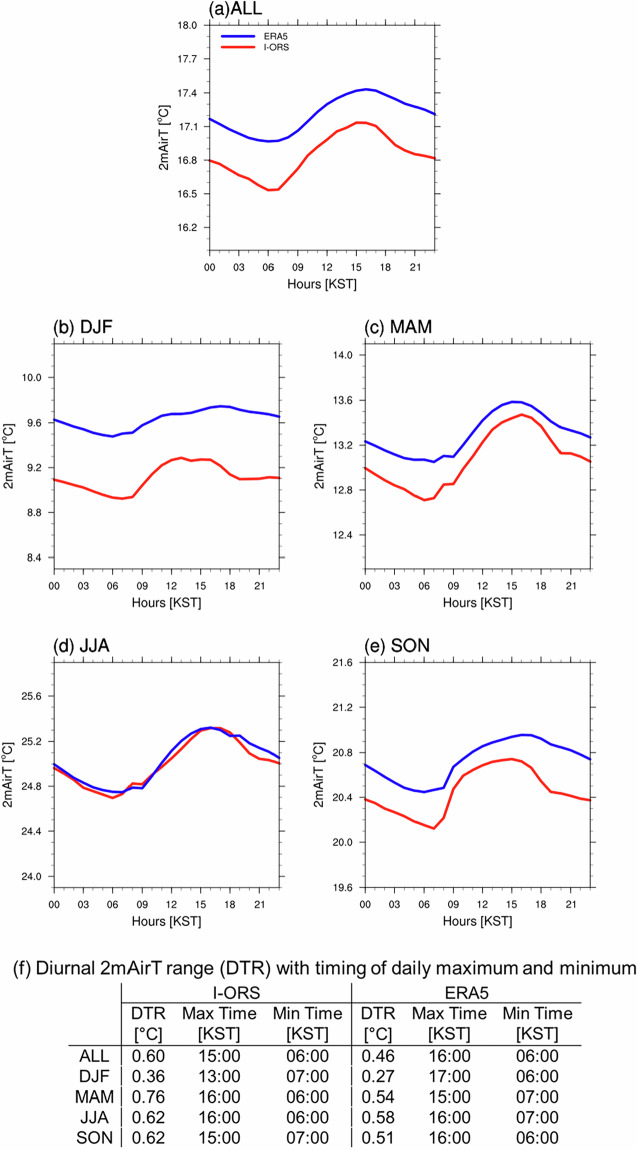
Diurnal cycle of 2 m air temperature (2mAirT, °C) for (**a**) annual mean (ALL), (**b**) winter (DJF, December–February), (**c**) spring (MAM, March–May), (**d**) summer (JJA, June–August), and (**e**) autumn (SON, September–November) during 2004–2023 from I-ORS (red) and ERA5 (blue). Panel (**f**) summarizes the diurnal 2mAirT range (DTR) and timing of daily maximum and minimum for each period. All times are in Korea Standard Time (KST = UTC + 9). ERA5 data are from the nearest grid point (32°N, 125.25°E) to I-ORS (32.12°N, 125.18°E).

To evaluate the dataset’s capability to resolve fine-scale temporal variability, we analyzed the diurnal cycle of 2mAirT using hourly I-ORS observations and ERA5 reanalysis data for the annual mean and individual seasons (Fig. 4). 2mAirT was selected for its pronounced diurnal cycle, with all times presented in Korea Standard Time (KST = UTC + 9) to preserve the physical relationship between temperature variations and local solar forcing. The annual mean diurnal 2mAirT range (DTR) at I-ORS was 0.60 °C, with daily maximum at 15:00 KST and minimum at 06:00 KST (Fig. 4a and 4f). ERA5 captured the overall diurnal pattern with a one-hour delay in maximum (16:00 KST) and same minimum timing, but with reduced DTR of 0.46 °C. Observed seasonal DTR varied from 0.36 °C in winter (DJF) to 0.76 °C in spring (MAM) (Fig. 4b–f). While ERA5 reproduced the seasonal modulation of diurnal amplitude (from 0.27 to 0.58 °C), it systematically underestimated DTR by 0.04 to 0.22 °C across all seasons. This underestimation has been reported over land surfaces^51^, suggesting a common limitation in ERA5’s representation of sub-daily variability. In terms of DTR magnitude, the strongest agreement between I-ORS and ERA5 occurred in summer (JJA), when both datasets showed comparable values (I-ORS: 0.62 °C; ERA5: 0.58 °C), whereas the largest discrepancy appeared in spring (MAM, 0.22 °C). Regarding diurnal timing, winter exhibited the most pronounced differences, with a 4-hour phase shift in maximum temperature timing (I-ORS: 13:00 KST; ERA5: 17:00 KST) alongside the weakest diurnal signal (I-ORS: 0.36 °C; ERA5: 0.27 °C). This winter timing difference likely reflects reduced solar forcing and increased synoptic-scale atmospheric variability^52^. These results demonstrate that ERA5 reasonably captured the observed diurnal timing (except in winter) but systematically underestimated the amplitude.

### Daily-scale reanalysis comparison

**Fig. 5:**
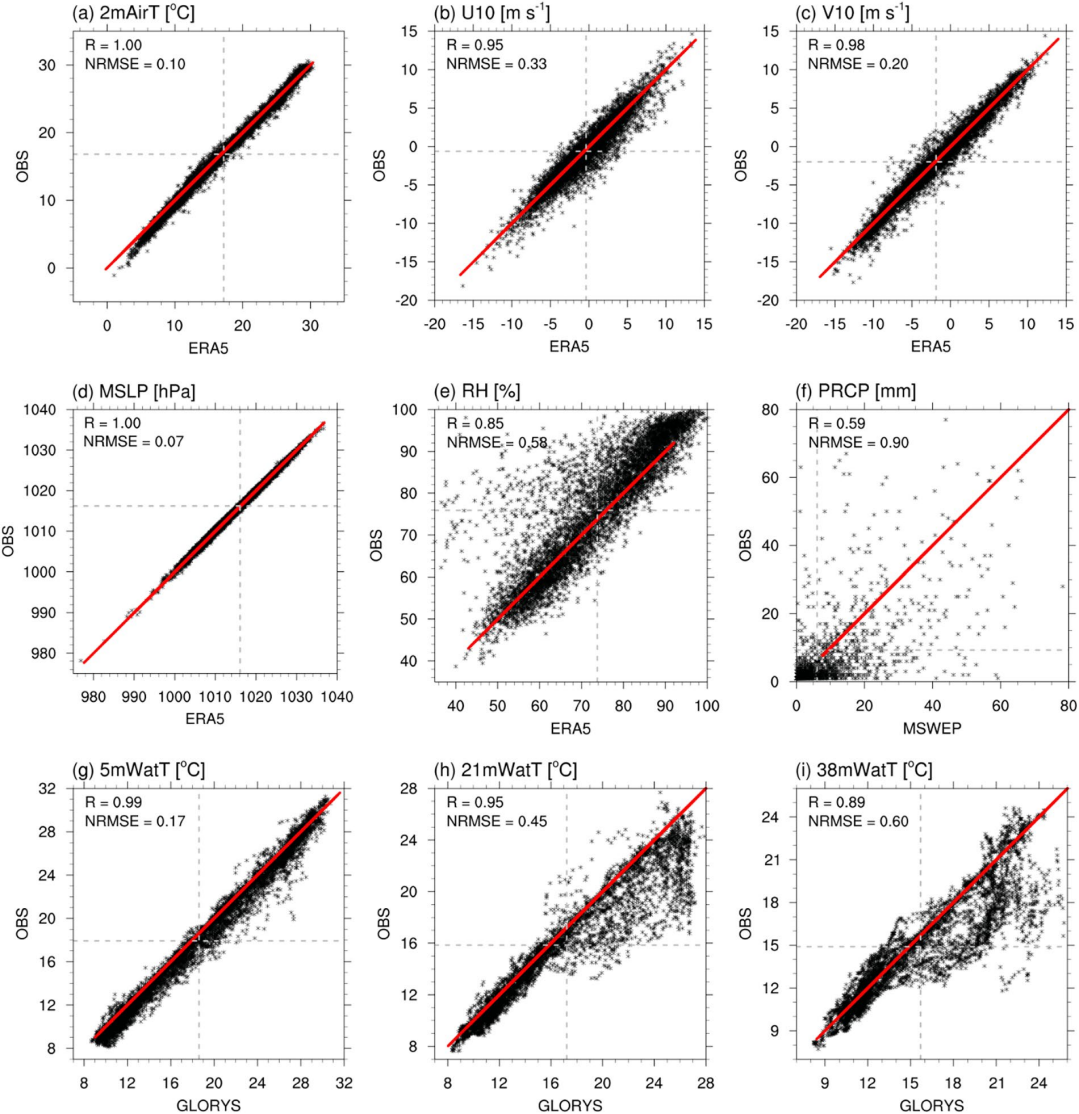
Scatter diagram of the relationship between multi-reanalysis (*x*-axis) and I-ORS (*y*-axis) daily data for the period 2004–2023: (**a**) 2 m air temperature (2mAirT, °C), (**b**) 10 m zonal wind (U10, m s^−1^), (**c**) 10 m meridional wind (V10, m s^−1^), (**d**) mean sea level pressure (MSLP, hPa), (**e**) relative humidity (RH, %), (**f**) precipitation (PRCP, mm), (**g**) 5 m water temperature (5mWatT, °C), (**h**) 21 m water temperature (21mWatT, °C), and (**i**) 38 m water temperature (38mWatT, °C). Each black asterisk represents daily data. Grey lines indicate the average for each variable, and the red lines represent the linear regression lines. Correlation coefficient (R) and normalized root mean square error (NRMSE) are shown in each panel. The values from the nearest grid points to the observation site—I-ORS (32.12°N, 125.18°E) —are used as follows: ERA5 at 32°N, 125.25°E; MSWEP V2 at 32.15°N, 125.15°E; and GLORYS12 at 32.083°N, 125.167°E.

Figure 5 presents a point-to-point comparison between I-ORS observations and reanalysis products from the nearest grid points using daily values. Statistical comparison between I-ORS observations and reanalysis products showed significant correlation coefficient (R) at the 99% confidence level, with low normalized root mean square error (NRMSE) values for most variables. The strongest agreements were found for MSLP (R = 1.0, NRMSE = 0.07), 2mAirT (R = 1.0, NRMSE = 0.1), 5mWatT (R = 0.99, NRMSE = 0.17), V10 (R = 0.98, NRMSE = 0.2), U10 (R = 0.95, NRMSE = 0.33), 21mWatT (R = 0.95, NRMSE = 0.45), 38mWatT (R = 0.89, NRMSE = 0.6), RH (R = 0.85, NRMSE = 0.58), and PRCP (R = 0.59, NRMSE = 0.9). These results suggest that reanalysis products successfully reproduced the daily variability observed at I-ORS, explaining between 35% and 100% of the total variance depending on the variable.

PRCP showed relatively lower correlation and higher error compared to other variables, which can be attributed to the lack of marine gauge observations^48^ and complex interaction among tropical, subtropical, and mid-latitude systems in East Asia^53^. In contrast to the good performance for 5mWatT achieved through satellite SST assimilation^46^, GLORYS12 reanalysis exhibited substantial overestimation—up to 10 °C—for subsurface temperatures (21mWatT and 38mWatT), with the largest discrepancies occurring primarily in September (not shown). This discrepancy indicates GLORYS12’s limited ability to reproduce the region’s strong vertical thermal structure, characterized by the pronounced summer thermocline overlying the Yellow Sea Cold Water Mass. Consistent with previous findings^54,55^, ocean reanalysis products that rely primarily on SST assimilation exhibit limited skill in representing subsurface temperatures during highly stratified summer periods. Additionally, such biases likely arise from the inability to represent sub-grid processes, such as turbulent mixing^56^, and to capture short-term variability associated with internal waves driven by tidal forcing and complex bathymetry^25,26,57^.

### Regional spatial representativeness

**Fig. 6:**
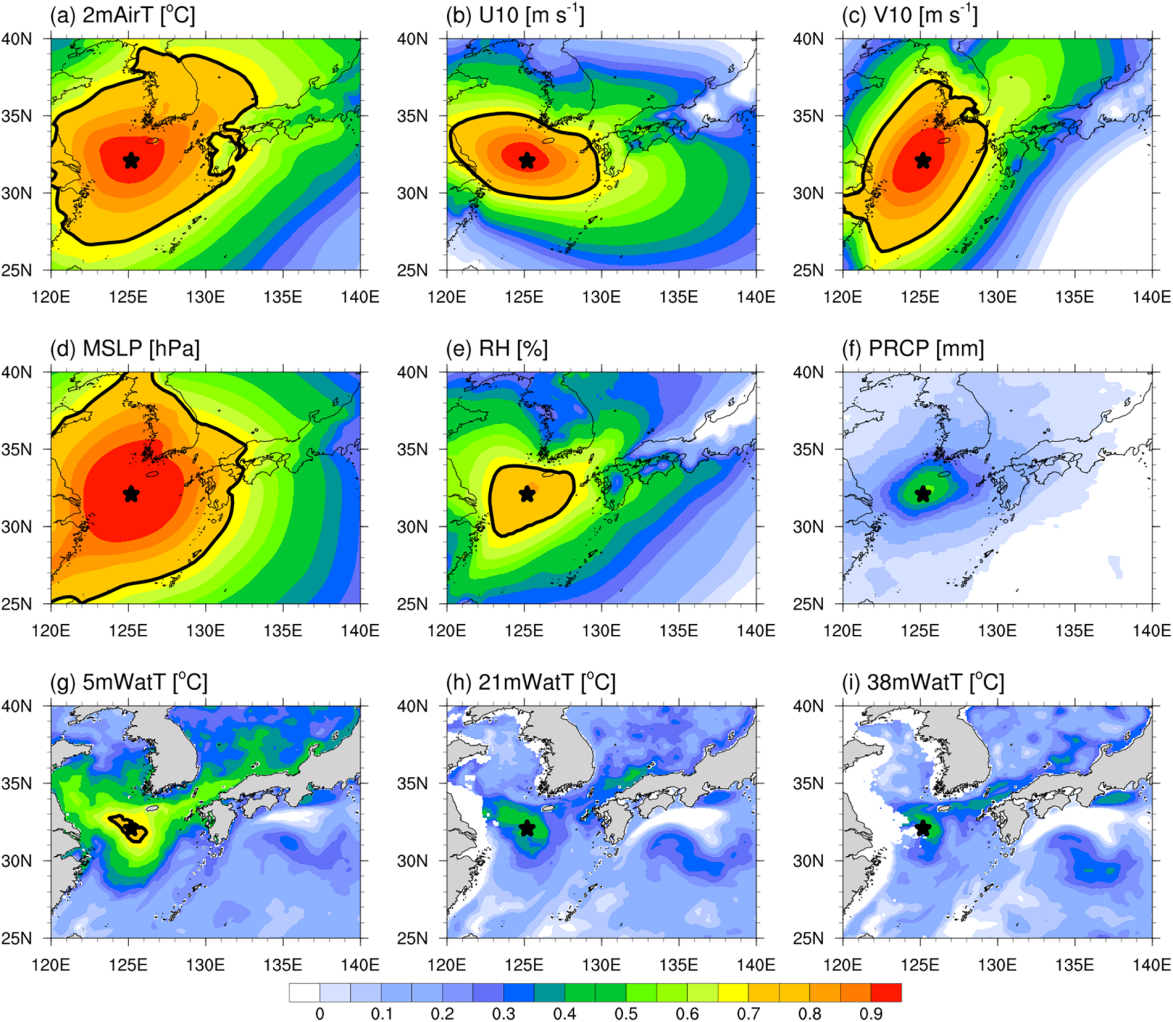
Spatial correlation map between one-point I-ORS (star marker) and multi-reanalysis daily anomaly data during 2004–2023: (**a**) 2 m air temperature (2mAirT, °C), (**b**) 10 m zonal wind (U10, m s^−1^), (**c**) 10 m meridional wind (V10, m s^−1^), (**d**) mean sea level pressure (MSLP, hPa), (**e**) relative humidity (RH, %), (**f**) precipitation (PRCP, mm), (g) 5 m water temperature (5mWatT, °C), (**h**) 21 m water temperature (21mWatT, °C), and (**i**) 38 m water temperature (38mWatT, °C). Daily anomalies are calculated by subtracting the 2004–2023 daily climatology. Black thick contours represent regions with correlation coefficients > 0.71, indicating areas where the I-ORS observations explain at least 50% of the local variability.

To assess the spatial representativeness of I-ORS point observations for the northern ECS, we plotted spatial correlation maps between I-ORS (base point) and regional reanalysis fields (Fig. 6). Daily anomalies were derived by removing the daily climatology (2004–2023). MSLP and 2mAirT parameters exhibited strong correlations (R = 0.65 and 0.61, respectively) over extensive sea and land areas of East Asia (25°–40°N, 120°–140°E). For these variables, regions where I-ORS data accounted for >50% (R > 0.71) of the observed variability extend ~500 km from the station. Wind components and humidity measurements (U10, V10, and RH) displayed moderate regional correlations within ~300 km of I-ORS (R = 0.42, 0.40, and 0.37, respectively). In contrast, PRCP patterns had the most spatially constrained relationship among atmospheric variables, with correlations limited to the immediate surroundings of I-ORS (30°–35°N, 121°–130°E, R = 0.25). Among oceanographic variables, 5mWatT exhibited moderate correlations (28°–38°N, 120°–135°E, R = 0.36) within ~120 km of I-ORS, encompassing the northern ECS, Yellow Sea, and Korea Strait. However, subsurface temperatures (21mWatT and 38mWatT) showed weaker correlations (R = 0.21 and 0.18) near I-ORS, similar to PRCP patterns. Interestingly, all water temperatures had fair correlations with the Kuroshio south of Japan (28°–31°N, 135°–139°E, R = 0.23–0.27) and moderate correlation with the Tsushima Current region (33°–36°N, 127°–135°E, R = 0.24–0.41), suggesting I-ORS could potentially capture signals from both the Kuroshio main stream and its branches.

### Long-term climate trend detection

**Fig. 7:**
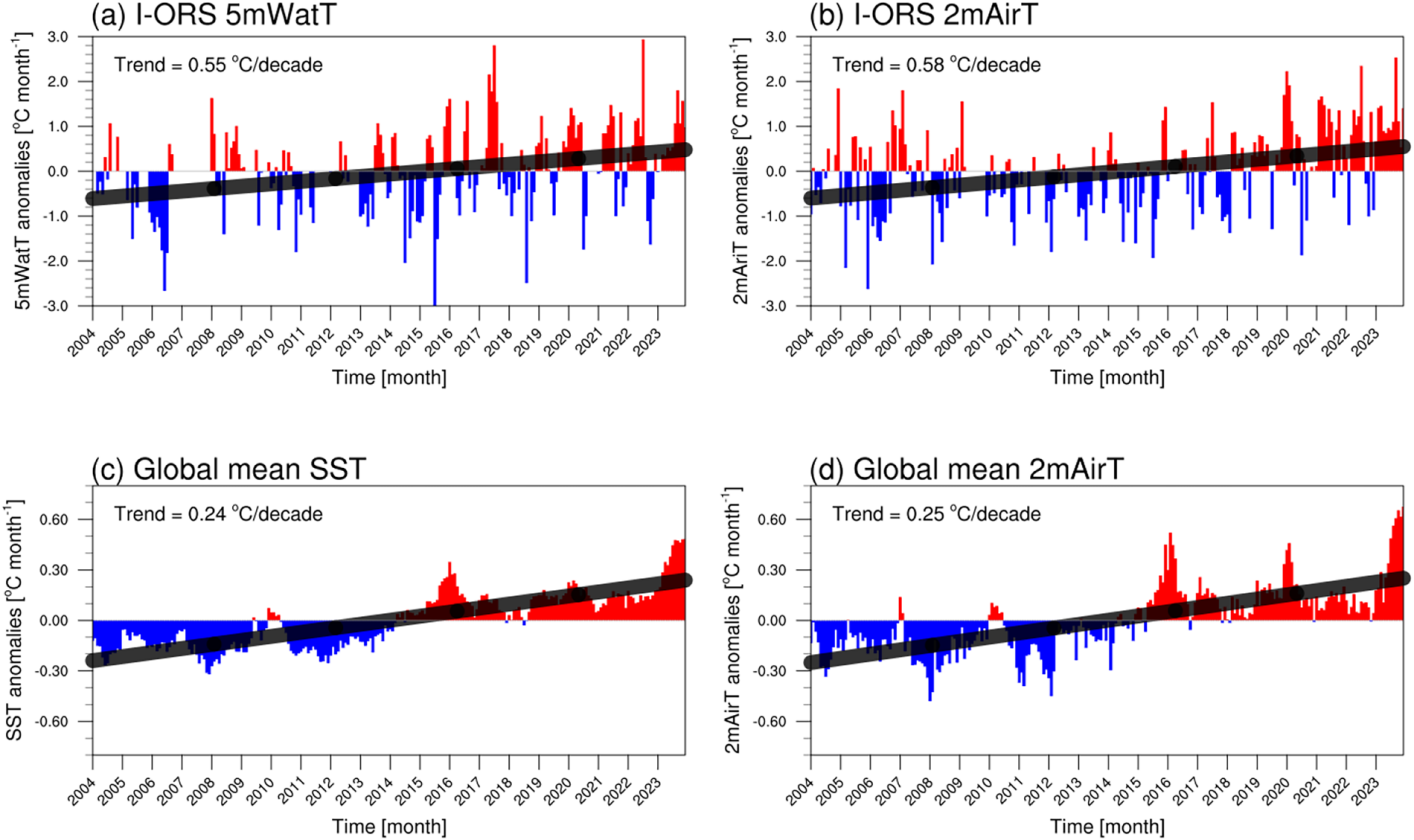
Time series of monthly anomalies of (**a,c**) near-surface temperature (°C) and (**b,d**) 2 m air temperature (2mAirT, °C) in (**a,b**) I-ORS and (**c,d**) the global mean (60°S–60°N). Red and blue bars represent monthly anomalies derived from reanalysis (OISST V2 in (**c**) and ERA5 in (**d**)) and I-ORS data, respectively. Black lines indicate the trend. Monthly anomalies are calculated by subtracting the monthly climatology (2004–2023), and trends are determined using linear regression methods.

Figure 7 displays the monthly anomaly time series from 2004 to 2023 for I-ORS and reanalysis data (OISST V2 and ERA5). Monthly anomalies were calculated by removing the 20-year climatological mean from each monthly value. Both 5mWatT and 2mAirT at I-ORS exhibited significant warming trends of 0.55 and 0.58 °C per decade, respectively, over the entire observation period. These rates were approximately twice the global average warming rates (60°S–60°N) during the same period (SST: 0.24 °C per decade, 2mAirT: 0.25 °C per decade). These results confirm that the northern ECS region is a climate change hotspot, with our long-term *in-situ* observations capturing the pronounced warming signals^1,4^.

## Data Availability

All I-ORS hourly datasets are openly available under CC BY 4.0 via the KIOST repository (10.22808/DATA-2025-2).

## Code availability

All data processing for I-ORS was conducted using MATLAB version R2024b. The related codes are publicly available in the KIOST repository at https://sciwatch.kiost.ac.kr/handle/2020.kiost/46771.
